# Disaster aid? Mapping historical responses to volcanic eruptions from 1800–2000 in the English‐speaking Eastern Caribbean: their role in creating vulnerabilities

**DOI:** 10.1111/disa.12537

**Published:** 2022-06-07

**Authors:** Jenni Barclay, Richie Robertson, Jazmin P. Scarlett, David M. Pyle, Maria Teresa Armijos

**Affiliations:** ^1^ Professor of Volcanology at the School of Environmental Sciences University of East Anglia United Kingdom; ^2^ Professor of Geology at the Seismic Research Centre University of the West Indies Saint Augustine Trinidad and Tobago; ^3^ Senior Research Associate at the School of Environmental Sciences University of East Anglia United Kingdom; ^4^ Professor of Earth Sciences in the Department of Earth Sciences University of Oxford United Kingdom; ^5^ Lecturer in Natural Resources and International Development at the School of International Development University of East Anglia United Kingdom

**Keywords:** disaster aid, Eastern Caribbean, hazards, Montserrat, recovery, response, Saint Vincent, volcanic eruptions

## Abstract

This paper uses volcanic eruptions on the Caribbean islands of Montserrat and Saint Vincent to explore the role that British colonial rule in the past and near past (1800–2000) has played in response to and recovery from hazardous events, and in turn, the influence that the nature of the hazards has on these responses. It shows that systemic vulnerabilities to natural hazards have been created by inadequate aid responses and longer‐term chronic problems and demonstrates that hazard impacts are compounded by them. Vulnerabilities could be reduced by analysing integrated hazard impacts to generate mitigative measures across hazards and identify actions that more closely match timescales of political decision‐making. Incorporating local knowledge and experience into risk analysis will enable the most effective use of aid resources, ahead of emergencies. Finally, coupling aid for long‐term development with emergency response would improve outcomes and adaptation to longer‐term vulnerabilities in immediate rebuilding and short‐term recovery.

## Introduction

**Figure 1:**
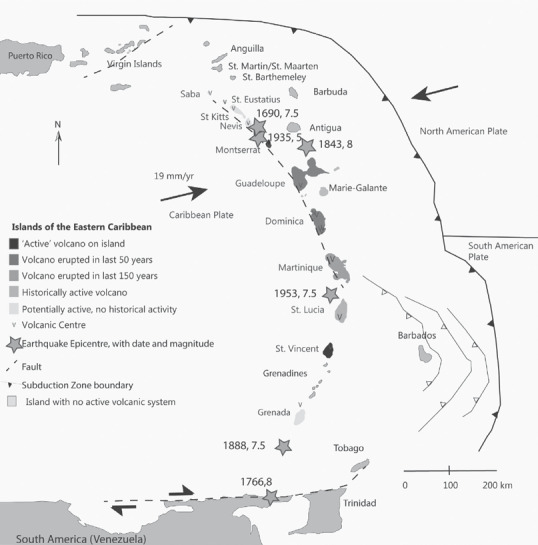
Key geographic, tectonic features and past tectonic and volcanic activity in the Eastern Caribbean **Notes**: ∗ Eruption dates and centres are from Lindsay et al. (2005), while block and plate boundaries and relative plate motion are from Smyithe et al. (2015). Black arrows denote plate motion. **Source**: authors.

The Eastern Caribbean consists of a largely volcanic chain of islands between 10^o^N and 20^o^N, straddling the boundary between the Atlantic Ocean and the Caribbean Sea (see Figure 1). Despite numerous hazards, the islands were attractive to explorers and then to colonisers due to their rich natural resource potential, primarily for growing sugarcane.

Several historical compilations of natural hazards exist. Their purpose is to document individual hazards and their impacts across the Caribbean (Robson, 1964; Lindsay et al., 2005; Mulcahy, 2008; Chenoweth and Devine, 2012), to chronicle the influence of multiple interacting hazards for single island states (Lewis, 1984; Boruff and Cutter, 2007; Wilkinson et al., 2016; Barclay et al., 2019a), and/or to understand the historical role of colonial governance in and around emancipation in shaping responses to hazardous events (Webber, 2018, 2019).

However, the extent to which relationships between topography, tectonics, governance, and markets have created conditions under which it becomes difficult to recover from the repeated and variable impacts of multiple hazards across the islands is relatively underexplored. Here, we use volcanic eruptions on the Caribbean islands of Montserrat and Saint Vincent to examine the role that British colonial rule in the past and near past has played in response and recovery to hazardous events, and the effect that the nature of the particular hazard has on these endeavours.

We assess how and why aid (both monetary and in‐kind) was requested, notably the conditions of its release and its influence on recovery. This allows us to analyse the extent to which economic, political, and social contexts affect the adoption of preparedness measures and their role in creating vulnerability to future events. Volcanic eruptions are typically agents of multiple hazards that create multiple impacts, but we also investigate the part played by other natural hazards in shaping the response to them or to subsequent unrest. Our longitudinal historical position allows us to understand how and why lessons were learned over time, or when mistakes were repeated, and what contributed to their repetition.

The aim of this paper is to identify practices and norms that had a negative bearing on capacities to cope with the challenges of an eruptive crisis, particularly those that appear resistant to the specificity of time and place. We use this to understand how vulnerabilities are created and amplified and how this recurrent cycle of risk creation might be broken. While risk is socially created, our integration of the physical aspects of eruptions into aid‐related responses makes it easier to discern the facets of hazard behaviour that encourage or lead to weak political or social responses or preparedness. So, a final goal is to determine how these historical lessons might be applied to improve response during and in anticipation of future hazardous events.

## A reflection on materials and methods: their influence on understanding vulnerability

This study utilised primary data from archived correspondence and administrative records. We examined declassified records of the Colonial Office, and original correspondence that made it into the ‘Blue Books’ for Saint Vincent, as well as contemporary newspaper articles (from electronic and national archives in the United Kingdom and Saint Vincent). In understanding historical responses to hazardous events, rich insights are gained from a thorough recording of the interactions and correspondence required of the colonial administration in the Eastern Caribbean. Since the Public Records Act of 1958 in the UK, most correspondence between colonial administrators, the UK government, and the (literate) population was kept and released to the National Archives, 50 and then 30 years after their writing.

However, it is comparatively rare to see direct records of the thoughts and motivations of all sectors of the population. In particular, the voices of those who were most adversely affected by hazardous events are rarely found in direct conversation, but rather are mediated and interpreted by landowners, line managers, administrators, and decision‐makers. Hence, we focused in particular on those records that provided glimpses into the views and actions of those sectors of the population that could not directly record their thoughts and opinions, including the original (uncensored) correspondence in the Blue Books, and correspondence files containing ‘offline commentary’ remarks and comments pencilled in the margins. Where possible, we used several different sources to corroborate any views and to fathom divergences of opinion. This is because our main goal is to understand the extent to which the actions of those with power and privilege created outcomes that mitigated any disaster in the short term, and whether they created or dissipated long‐term future risk by changing vulnerabilities (economic, social, physical, or systemic) for *all* of the population. We also draw on the work of other historical scholars of the Caribbean who have attempted to comprehend the influence of politics and prejudices brought to bear in documentation on the lived experiences of indigenous peoples, the African‐Caribbean and indentured population in the Eastern Caribbean (see, for example, Richardson, 1992; Honychurch, 2017; Webber, 2018, 2019). While we recognise that our analysis of vulnerabilities using the historical record is not complete, by concentrating on multiple sources that specifically document both physical and wider social impacts, we try to acknowledge the partisan viewpoints and the limits to interpreting the data, including the prejudices applied in the original documentary evidence. A full list of our archival sources is provided in the Appendix.

We begin by considering the trajectories of the social, cultural, and political context, and the near continuous interruptions arising from all hazard events. We use this to compare the detailed responses to four volcanic eruptions separated by more than 180 years. And then we use this to illuminate the enduring negative influences on capacities to cope with and recover from hazardous events and appraise how these might be addressed in the future.

## Analysis

### Political context during the nineteenth and twentieth centuries: governance, infrastructure, and the creation of vulnerability

The English (later the ‘British’ after the Act of Union in 1707) began to settle in the Lesser Antilles from 1623 when Thomas Warner began the Colony of Saint Christopher (Richardson, 1992). The momentum for a permanent settlement came from the production of agricultural crops for export, trade, and profit. This remained the main motivation behind further British settlement and development throughout the Lesser Antilles, amplified by the need to demonstrate colonial power and tempered by territorial skirmishes, principally with French colonisers. ‘Possession’ of these islands was uneven in time and space (see Figure 1 and Table 1). Seventeenth‐century colonies were established in Saint Kitts in 1624, Nevis in 1628, and Montserrat and Antigua in 1632 (Beckles, 1998).

**Table 1:** Key changes in governance and colonisation for Montserrat and Saint Vincent∗

	**Montserrat**	**Saint Vincent**
Indigenous population (island name)	Caribs (Alliougana)	Kalinago (Hairouna) and Garifuna
British colonisation	1632	1763
Other colonising forces	France, 1660; 1782–83	France, 1719–63; 1779–83
Independence	UK Overseas Territory	1979
Governor, nominated Council, elected Assembly	1971–present	1800–33; 1969–79 (as associated state of the UK)
Federation (Windward or Leeward Islands)	Leeward Islands Colony (1671–1833); British Leeward Islands (1833–1958); West Indies Federation (1958–1962)	British Windward Islands (1833–1959); West Indies Federation (1959–62)
Crown Colony (largely unelected representation)	Part of the Leeward Islands Crown Colony	1877 (Legislative Council, established in 1925)
Universal suffrage	1951	1951

**Source**: authors, with administrative timings from Banton (2008).

Some of these colonies briefly changed hands with the French because of Anglo‐French Wars, but primarily they remained in British hands (see Table 1). By contrast, Saint Vincent and the Grenadines, Dominica, Grenada, and Saint Lucia were earlier French colonies—where some authors have inferred a less aggressive relationship between the invading settlers and the indigenous population (Stinchcombe, 1996; Honychurch, 2017)—but changed hands in the late eighteenth century as ‘ceded’ territories (for instance, in 1763, Saint Vincent, Grenada, and Dominica passed into British hands, and Saint Lucia into French hands) or ‘consolidated British territories’ (such as Saint Kitts in 1783 as part of the Treaty of Versailles), and (again) as a final outcome of the Anglo‐French Wars that ended in 1815.

The apparent political stability of 1800–2000 was offset by frequent changes in governance arrangements and the long‐term consequences of an economic model that relied on enslaved and indentured labour, followed by slow social change in response to emancipation in 1834 (summarised in Table 1). Tensions between resident settlers and the British colonial government, complicated by absentee landowners (resident in the UK but dependent on island profitability), are well documented elsewhere (see, for example, Sires, 1957; Murdoch, 1984; Richardson, 1992; Gibson, 2014). In the context of hazardous events, one of the critical outcomes of these tensions is changes to governance structures. These controlled which groups possessed the agency to act (and on behalf of whom), and on what timescale in response to hazardous events. By the beginning of the nineteenth century, each of the islands typically possessed an unelected Governor and a nominated Council (representing and acting on behalf of the UK monarch and government) and an elected Assembly (entirely composed of high‐status settlers, with independent money‐raising powers). The quality of governance varied and had strong impacts on the efficacy of this system.

Differences of opinion about the use of enslaved labour between the ‘liberal elite’ in the UK (where more individuals or interest groups were strongly minded to support emancipation) and the ‘planter classes’ (Richardson, 1992) domestically and in the UK meant that there was generally a move to tighten remote control of island governance, increasing unelected representation and decreasing local free citizens’ capacity to make choices regarding central budgets as mistrust grew between these groups (Stinchcombe, 1996). Power and money were thus disseminated through federated governance and the establishment of ‘Crown Colonies’ (with local Administrators reporting to the Governors responsible for several islands, who in turn represented the interests of the UK) (see Table 1). We will explore the important role of this structure in the timing and nature of aid in the face of current and future hazard events.

Through the late nineteenth and early twentieth centuries, there was some recognition of the need for improved living and economic conditions and self‐governance on the islands. Following the First and Second World Wars, the drive for independence increased as a democratic response to universal suffrage (see Table 1). This prompted some changes that created a system that was preparing itself for economic independence and self‐governance. But again, the efficacy of the system at the time has been the subject of critical scrutiny; for example, Stinchcombe (1996, p. 13) notes that: ‘the racist oligarchic parliamentarianism of the British islands, backed by a more or less non‐governing Colonial office, provided stability and some civility, but its tutelary democracy had provided more tuition than democracy, and not much of either’. The islands examined here briefly became part of the West Indies Federation in 1958 and then Saint Vincent became independent in 1979. Montserrat remains a UK Overseas Territory to this day.

### Social and cultural context during the nineteenth and twentieth centuries: circulation of ‘aid’, land entitlements, and creating vulnerability

The troubled relationship between land, ownership, and profit emerged at an early stage of Caribbean colonisation in Montserrat with the establishment of a ‘planter society’ by the mid‐seventeenth century (Fergus, 2001), largely drawn from Irish or Anglo‐Irish elites (Ryzewski and Cherry, 2015). Sugar profitability peaked between 1730 and 1760, fuelling a rapid rise in an enslaved population taken from Africa, and driving an increase in absentee plantation owners, determined to enjoy their wealth and seek power by living in Great Britain (Ryzewski and Cherry, 2015). A small but significant underclass of farmers and labourers comprised freed slaves, lower class Irish (and English) citizens, and predominantly white Catholic refugees who had fled the consequences of the English Civil War (1642–51) and then the American War of Independence (1775–83). Typically, this group was only able to purchase marginal agricultural lands in and around the capital of Plymouth, now buried under volcanic deposits. By 1811, some 94 per cent of the local population were enslaved African labourers, although the sugar industry had been destabilised by a combination of market forces, natural hazards, and crop and human disease. Absentee landowners became indebted and living and working conditions were dominated by endemic labour and food shortages and the response of plantation managers to generate profitability for their owners despite these challenges.

Saint Vincent's history of British occupation began with secession from France in 1763. The island was surveyed, and parcelled up for sale and profit, with little regard for current occupancy. The assumption was that infrastructure and healthy local governance would be an outcome of that profit, essentially trying to reapply a model that had been somewhat successfully enacted on other islands (Barclay et al., 2019a; Scarlett, 2020). British occupation of and encroachment on the marginal agricultural lands led to the First (1769–73) and Second (1795–97) ‘Carib Wars’. The Garifuna were then largely exiled to present‐day Honduras. Not long after the Second Carib War, plantations near and around Georgetown began to appear in the sugar production records. Nonetheless, in contrast to Montserrat, Saint Vincent still had a small but significant indigenous population at the start of the nineteenth century, albeit largely confined to marginal lands near high‐risk volcanic areas, deliberately removed from potentially profitable areas and colonial infrastructure.

Thus, by the early nineteenth century, hazard response was embedded in a hierarchical system of landowners and administrators who were themselves governed by the UK. Fundamentally, landowners were indebted, and the majority of the population possessed little or no agency to respond on their own behalf or articulate their needs. In the ceded territories, land had usually been procured via a government loan (Murdoch, 1984). And in Montserrat, debt was also accumulating. The mountainous terrain of the volcanic islands was not as well‐suited to large‐scale profitable monoculture to repay debts. Subsequently, when hazardous events occurred, the economic shock associated with the destruction of infrastructure and crops created responses that looked outwards for aid and financial assistance directed towards the needs of landowners. Little attention was given to the needs of other groups or communal infrastructure, as requests for help were channelled through local landowners and dependent on UK intervention centred on major infrastructure or post‐disaster responses. In turn, financial aid to individual landowners was provided as loan‐in‐aid by the UK government at a crippling interest rate of four per cent (Barclay et al., 2019a).

Following emancipation in 1834, the Black majority on the islands did not secure sustained access to land and independent livelihoods. They were forced to settle and work on less fertile land that was more exposed to hazards (Ryzewski and Cherry, 2015; Barclay et al., 2019a; Ryzewski, Cherry, and McAtackney, 2019), often on steep slopes on the edge of larger plantations nearer active volcanoes. By the late nineteenth century, this system of debt, poverty, and weakening infrastructure had created endemic poverty in the Caribbean, such that Joseph Chamberlain, the then Secretary of State for the Colonies, referred to it as ‘the Empire's Darkest Slum’ (cited in Richardson, 1992).

Disaster relief is considered to be the provision of humanitarian aid that alleviates impacts and losses due to a catastrophic event, usually seen as an emergency response. In the nineteenth century, UK colonialism and humanitarianism had a complicated relationship (Barnett, 2011). There was evidence of some assumed responsibility for the state of affairs described above in the late 1800s in the form of repeated state‐driven inquiries, in response to both endemic poverty and hazard‐driven losses (House of Commons, 1855; Morris, 1897; Naftel, 1898; West India Royal Commission, 1897, 1898). There was also a proliferation of charitable works and welfare associations, echoing UK domestic practices and views on vulnerability and who or what ‘deserved’ relief.

However, the fundamental response of the UK government (bilateral aid) and charitable associations (voluntary aid) was still driven by the need to preserve social hierarchies, a belief in the ability of markets to create economic stability, and a paternalistic need to solve the problem *as perceived by the donor* rather than that of the benefactor. Furthermore, at this time, there was an emerging view that public institutions were superior to private charity, tending to push responses into the hands of the state (Barnett, 2011). Following hazardous events, private and state donations were frequently compiled and directed to the ‘Mansion House Fund’ (a centralised source of relief funds and donations established by the Lord Mayor of London), after which funds were dispersed via ‘The Crown’, channelling voluntary aid via multilateral investments.

Consequently, stereotypes and fundamentally racist views drove decision‐making ‘in the best interests’ of the most vulnerable communities (Webber, 2018, 2019). By the turn of the twentieth century, the circulation of aid was still rooted in the premise by which the islands had been colonised by the British. Landowners were not only able to access aid funds, but they were also able to apply for temporary tax exemptions (such as Land Tax Relief) and still found means to have accumulated debts written off. By contrast, the Black majority on Saint Vincent had no mechanism to request the form or size of aid. Power differentials affected access to the decision‐making process concerning how donations were spent, and choices that could be made in the face of vulnerabilities generated by the hazardous events. Historical analysis of philanthropy (Henry, 2008) has identified at this time the rise of community‐based practices in the region, including ‘susu’ (communal savings to be spent in a time of need), and a tradition of mutual self‐support, supplemented by donations of cash and goods from those who had migrated overseas.

Following several severe hurricanes in the 1920s and the economic impacts of the Great Depression (1929–39), the Caribbean remained in systemic poverty by the 1930s, triggering further state‐sponsored analyses (interrupted by the Second World War of 1939–45) (Orde Browne, 1939; West India Royal Commission, 1945). This time, the longer‐term evidence gathering by the West India Royal Commission attempted to focus on prospective practices to dismantle vulnerabilities such as education and capacity‐building. The recommendations were still influenced by market‐driven economics—this time concentrating on bananas as a single potentially profitable crop (Thomson, 1990)—and the need to respond to an emerging local and UK push for independence.

A positive exemplar of the value of longer‐term investment in this period is the creation of the Seismic Research Unit (now known as the Seismic Research Centre (SRC)) from a ‘colonial development and welfare’ project, marking the very first emergence of a proactive approach to hazard preparedness and monitoring rather than a reactive response to the devastation left behind. The SRC is the agency responsible to this day for monitoring earthquakes and volcanoes in the English‐speaking Caribbean. Nonetheless, it has been plagued throughout its history by unstable and inadequate funding, with core resources driven by the continued occurrence of geophysical hazards in the Caribbean (Joseph et al., 2022),^2^ reminding the government of the value of a monitoring agency.

Offsetting these state‐driven initiatives was the fact that the emerging (state independent or international) humanitarian sector remained comparatively inactive at this point in the Caribbean, despite the need generated by continuous hazard events (see Table 2) coupled with the widespread poverty created by the historical and recent past. This sector (comprising, for instance, the Red Cross and the Pan American Health Organization) strengthened through the latter part of the twentieth century, initially facilitated at the grassroots level by strong networks offered by various Christian churches (Webson, 2010). However, even in the past few decades, long‐term approaches have proved difficult for these organisations, limited by the duration of multilateral international funding that fuels initiatives or the enthusiasm of private individuals to continue to give as the memory of triggering events fades.

**Table 2:** Decision‐making and budget‐holding departments requiring inputs for the movement of disaster aid: four focus eruptions

	**Soufrière Saint Vincent, 1812**	**Soufrière Saint Vincent, 1902**	**Soufrière Saint Vincent, 1971, 1979**	**Soufrière Hills Volcano, Montserrat, 1995–97**
UK departments	Colonial Office, Treasury	West Indies Atlantic Department, Colonial Office, Colonial Office, Treasury	Ministry of Defence, Home Office, Treasury, West Indies Atlantic Department, FCO (Overseas Development Agency)	Ministry of Defence, Home Office, Treasury, West Indies Atlantic Department, FCO, Department for International Development (emergency aid), Department for Education, Department for Health
Caribbean departments	Appointed Assembly	Leeward Islands Governor	Elected Assembly, Prime Minister	Elected Assembly, Chief Minister (UK Governor)
Equivalence with other hazard events	Yes	Yes	Yes	Yes
Other aid‐awarding entities	–	Mansion House Fund, other bilateral funds (notably from Canada and the US)	European Union, Red Cross, United Nations agencies, church bodies, other bilateral funders	European Union, Red Cross, United nations agencies, church bodies, other bilateral funders

**Source**: authors.

### Continuous hazardous events in the nineteenth and twentieth centuries

**Figure 2:**
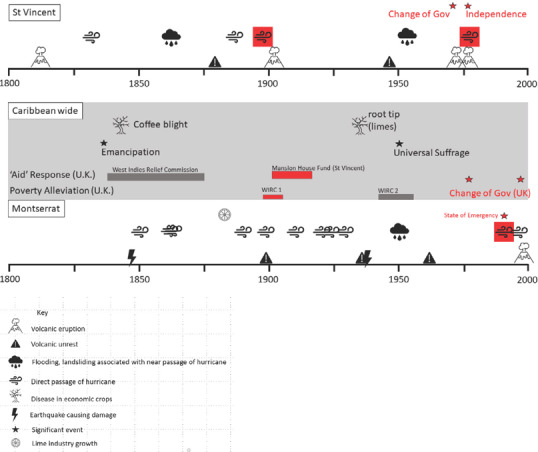
Island timelines with hazards above each island timeline∗ **Notes**: ∗ the middle panel shows relevant initiatives and events that impacted response and recovery. The events surrounded by boxes are the ‘index’ events influencing the response to case study eruptions. ‘Poverty alleviation’ measures are in response to extreme deprivation across several islands in the Eastern and wider Caribbean. WIRC refers to the strategies and initiatives developed in response to the two West India Royal Commission reports on this poverty. The aid measures are described in more detail in the main text. **Source**: authors, based on the NOAA's (National Oceanic and Atmospheric Administration) National Hurricane Centre Data Archive (https://www.nhc.noaa.gov/data/) and the UK's National Archives (https://www.nationalarchives.gov.uk/), as well as the following works: Lindsay et al., 2005; Chenoweth, 2006; Wilkinson, 2015; Pyle, Barclay and Armijos, 2018; Barclay et al., 2019b).

Figure 2 illustrates the timeline of hazardous events in the context of other environmental challenges and sociopolitical changes across the two islands. In their compilation of historical cyclones in the Eastern Caribbean, Chenoweth and Devine (2012) observe a 50‐to‐70‐year cycle in ‘accumulated cyclone energy’, probably associated with multi‐decadal variations in North Atlantic sea surface temperature, and the Atlantic multi‐decadal oscillation. These translate into periods of lower cyclone activity across the region and on any one island. This variance is around the timescale of a human lifetime but much greater than that of a political cycle; it can be seen as periods of relative meteorological calm in our datasets (see Figure 2). There is a conspicuous role for accumulated vulnerability due to the impacts of repeated storms, or storms combined with other risks in a relatively short space of time during periods of high energy. This threat will increase in the future as a warmer climate and consequently seas will act to increase the severity of hydrometeorological events (Stephenson et al., 2014).

A further significant non‐eruptive hazard event that impacted Montserrat was the Guadeloupe earthquake of 1843. Strong ground shaking effectively wiped out the local island infrastructure on Montserrat (Ryzewski and Cherry, 2015), affecting sugar production for several years afterwards. The close timing relative to the social upheaval associated with emancipation meant that this contributed to economic and social vulnerability for several decades. Montserrat was still recovering from the impacts of Hurricane Hugo (1989) when the Soufrière Hills stratovolcano began to erupt in 1995. As for Saint Vincent, two volcanic eruptions have a conspicuous (non‐causal) association with impactful hurricanes: Hurricane Allen in 1980 and the Windward Islands Hurricane in 1898.

Furthermore, in the mountainous topography of the Caribbean volcanic islands, the enhanced rainfall associated with any one hurricane season usually generates shorter‐term local challenges (such as road and building or localised crop damage) associated with localised landsliding and flooding. Drought, too, has a role to play in amplifying vulnerabilities to disease and resource shortage. In the following subsection, we deepen the analysis by focusing on specific volcanic eruptions.

### Mapping historical responses to volcanic eruptions through social, cultural, and political changes: the creation of volcanic risk

Our exemplar eruptions capture four significant time periods: the period during which slavery was in operation (1812, Saint Vincent); the islands after several decades of emancipation (1902–03, Saint Vincent); preparing for independence (Saint Vincent, 1971–72 and 1979); and a near historical response (Montserrat, 1995–97). We use a detailed analysis of the correspondence around these eruptions to understand the precise motivations and drivers of the distribution of aid described more broadly above. We also now consider the role that capacity to anticipate eruptions and likely impacts played in moderating preparedness, as well as the impetuses of risk exposure in each period.

#### The 1812 eruption on Saint Vincent

This eruption was not anticipated and generated several pyroclastic density currents and lahars into river valleys during the period from 27 April–6 May 1812 (Anderson and Flett, 1902; Huggins, 1902). Preliminary explosions led to a climatic eruption from 30 April–1 May. Activity subsided after that, although there is some written evidence of a minor pulse of activity on 8 June. This caused widespread disruption to those in the north of the island, including many large plantations. The post‐eruptive morphological change in the Wallibou Valley in particular increased the likelihood of flash flooding, which resulted in further loss of life some years after the eruption.

The archival records of the 1812 eruption on Saint Vincent demonstrate the attitudes towards aid typical of this era (see the ‘social and cultural context’ subsection above). Contemporary scientific knowledge afforded no opportunity for responsive aid, only reactive aid (merely a few recorded warning signs of activity, just a few days before) (Anderson and Flett, 1902). The immediate need for food in the aftermath was met by donations from nearby colonies and the purchase of supplies overseen by an internal committee. In keeping with practice at that time, damage was estimated to infrastructure and to economic crops but not to people (Smith, 2012; Scarlett, 2020). Financial aid requests came from the Governor on behalf of estate owners, and from the estate owners themselves. Thus, on an island with a total population of 26,740 (more than 90 per cent enslaved) (Smith, 2011), only some 12 individuals (all landowners) were compensated as an expression of aid (Scarlett, 2020), although 802 people were evacuated and 53 were recorded as killed. The articulation of financial loss implicitly mitigated deaths and damaged livelihoods via compensation for lowered productivity relevant to the wealthy landowners, rather than by direct attempts to improve recovery for individuals, families, and communities.

In addition, there is some evidence that this aid was manipulated for personal gain by these groups. To illustrate, a disproportionate sum was awarded to a larger estate further from the locus of destruction (The Gazette Office, 1825). In this instance, this money was probably used to compensate a landowner for his land being used to rehouse displaced enslaved people (see the Appendix).

The most exposed populations were the indigenous Kalinago and indigenous‐African communities (the Garifuna), settled on marginal lands very close to the volcano. Contemporary accounts record them as abandoning their homes and livestock (near Morne Ronde) and fleeing ‘precipitately’ towards town on the morning of 30 April, but casualties in this group are not recorded.

Webber (2019) argues that the occurrence of a disaster in the nineteenth‐century English‐speaking Caribbean created an opportunity to challenge the social distance and racial hegemony that underpinned colonial rule. In the case of this disaster, though, the structures in place during enslavement quashed that opportunity. From his demographic and economic survey of plantation recovery, Smith (2011) shows that the relief provided facilitated, in fact, a rise in plantation populations in the highest volcanic hazard zone following this eruption. Long‐term mitigative actions, such as via changing agricultural practices or land use, or changes in the trajectories of risk creation were not encouraged by the disaster relief.

#### The 1902–03 eruptions of Soufrière Saint Vincent

Although warning signs prior to this eruption were recognised up to a year in advance, particularly by the indigenous population (Pyle, Barclay, and Armijos, 2018), the administration was distracted by continued landsliding and rebuilding as a consequence of the hurricane of 1898, so there is no pre‐emptive discussion of the unrest, let alone response, in the official records. After some initial explosions on 6 May 1902, the initial and largest series of explosions reached a climax on the afternoon of 7 May, subsiding overnight. There were further explosive eruptions in May, September, and October 1902, and one final explosive event in March 1903. The eruptions on 7 May 1902 sent destructive flows into most areas of the current ‘red’ zone of Saint Vincent, leading to profound damage and severe loss of life—it is estimated that 1,565 lives were lost on the Windward side of the island) (Blue Book, 1902). The most exposed populations were those living in marginalised lands near the volcanoes (indigenous communities and small‐scale subsistence farmers). On the morning of 7 May, among the workers on the plantations on the Windward side of the island, a normal working day had commenced (Pyle, Barclay, and Armijos, 2018).

With such profound damage and loss of life, aid was rapidly mobilised in response to the devastation. Relocation, rehousing, and rebuilding were needed owing to these eruptions. Rapid communications regarding ongoing activity and damage reports were facilitated by experience of the recent hurricane (Anderson and Flett, 1902). Nonetheless, socially and politically, this eruption served to reinforce the status quo (with large landowners retaining a tight grip on land use) and amplify or accelerate processes of migration and bankruptcy already in train, where landowners were failing to make effective use of these lands (Pyle, Barclay, and Armijos, 2018).

A significant change from 1812 was more clear articulation of estimates of loss of life and damage to the livelihoods of more than just the landowning minority. Aid was articulated around the needs of the most vulnerable and did not just manifest as grant‐in‐aid loans. Relief from Great Britain and its colonies, as well as from other foreign nations through sources (such as governmental, authorities, royal or community entities), was substantial for this eruption. These donations, along with other funds related to relief efforts, were amalgamated into a single pot created by the colonial government called the ‘Soufrière Relief Fund’, administered by the (UK‐based) Mansion House Fund.

However, the economic model that permeated island governance (see the ‘political context’ subsection above) still meant that the circulation of aid was rooted in the premise by which the islands had been colonised by the British. Landowners were not only able to access aid funds, but they were able to apply for temporary tax exemptions (such as land tax relief), and they still found ways to have accumulated debts written off. Power differentials affected access to the decision‐making process concerning how donations were spent and choices that could be made in the face of vulnerabilities generated by the repeated eruptions of 1902–03. The most frequent direct beneficiaries were often absentee estate owners. Details of expenditure and correspondence (see, for example, Webber, 2019; Pyle, Barclay, and Armijos, 2018; Scarlett, 2020) demonstrate that power held by the large landowners created a perception among themselves and the colonial government that they were the ‘real’ sufferers and that supporting them also offered the best route to recovery (see the Appendix).

This patrimonial view of landowners towards their labourer population and their continued belief that monocultural (sugar and arrowroot) cultivation was still the most prosperous route to recovery persisted despite evidence on the decline of these crops (Pyle, Barclay, and Armijos, 2018). Aid framed in terms of the provision of labour on behalf of the majority population was a pervasive viewpoint; a lack of compliance with schemes devised on behalf of that population was interpreted as a manifestation of indolence (Webber, 2019). Considerable pressure was exerted to solve immediate problems via migration, and neighbouring administrations were encouraged to make offers of labour.

There is no archival evidence that peasant landowners were directly able to access special funds (either for the 1898 hurricane or the 1902–03 eruptions) or that they were able to access the tax exemptions. However, they were offered ‘doles’ (weekly relief) (Pyle, Barclay, and Armijos, 2018) and ‘Compensation for loss of Live Stock … without any conditions’ (despatch by Windwards Governor, Robert Baxter Llewelyn, 5 January 1903; Blue Book, 1903), albeit with caveats attached for working‐age males (see the Appendix). The coincidence of the 1898 hurricane and the 1902–03 eruptions and the longer‐term development effort arising from the West India Royal Commission's report of 1897 meant that recovery was assisted by the land purchase scheme—which sold five‐acre plots and emphasised small‐scale agriculture ‘by establishing a solid nucleus of peasant proprietors with a variety of cultivable products, [which] will destroy that too exclusive devotion to particular industries which has proved the bane of the Island’ (Colonial Reports, 1901)—and the creation of new, permanent settlements on Saint Vincent. However, this was confused by a lack of trust between the majority Black population and local authorities and the authoritarian requirement to choose crops cultivable for cash.

Later, in March 1903, the final explosive eruption of La Soufrière rendered many lands once again uninhabitable. Yet, wrangling persisted over the selling of land, the creation of new settlements, and other long‐term solutions to help islanders recover in the way that they wished, rather than migrate as indentured labour to estates on neighbouring islands. The continued explosions associated with the year‐long eruptive period meant that Georgetown and surrounding villages were progressively abandoned, as living with the large quantities of ash became difficult.

The coincidence of the eruptions and the broader rebuilding programme cemented a need to place most of the infrastructure and capacity in the south of the island. Several of the ‘resettlement’ sites developed in the longer term into villages. At these sites, economic activity was lower and levels of deprivation were higher than in previously established settlements. This uneven development remains to this day. Thus, in the context of the 1902–03 eruptions, the inadequate longer time frame (years) responses to recovery have created conditions that persist more than a century later.

#### The 1971–72 and the 1979 eruptions on Saint Vincent

**Figure 3:**
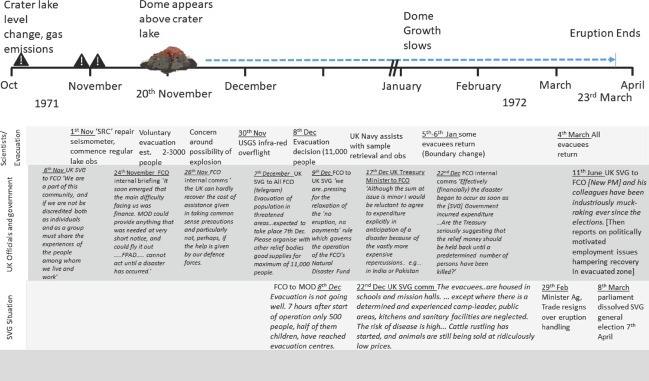
Synthesis of volcanic, scientific, political, and social activities during the 1971–72 eruption of La Soufrière, Saint Vincent **Notes**: All materials and timings sourced from original letters and correspondence in National Archive FCO 63 1022. 881, 882, and 883 and contemporary scientific descriptions (Aspinall, Sigurdsson, and Shepherd, 1973). Abbreviations are as follows: SVG—Saint Vincent and the Grenadines; FCO—Foreign and Commonwealth Office; UK SVG—UK representative based in Saint Vincent and the Grenadines; MOD — UK Ministry of Defence; SRC — Seismic Research Centre, University of the West Indies (then known as the ‘Seismic Research Unit); USGS—United States Geological Survey; and PM SVG—Prime Minister of Saint Vincent and the Grenadines. **Source**: authors.

**Figure 4:**
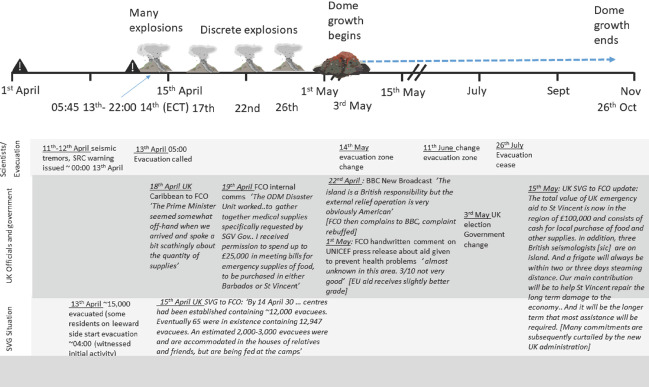
Synthesis of volcanic, scientific, political, and social activities during the 1979 eruption of La Soufrière, Saint Vincent **Notes**: all materials and timings sourced from original letters and correspondence in UK National Archive files FCO 44 2031 and FCO 44 2030 and contemporary scientific descriptions (Shepherd et al., 1979). Abbreviations are as follows: SVG—Saint Vincent and the Grenadines; FCO—Foreign and Commonwealth Office; UK SVG—UK representative based in Saintt Vincent and the Grenadines; BBC—British Broadcasting Corporation; SRC—Seismic Research Centre, University of the West Indies (then known as the ‘Seismic Research Unit); PM SVG—Prime Minister of Saint Vincent and the Grenadines. **Source**: authors.

The historical records of the administrative process regarding the 1971–72 and the 1979 eruptions on Saint Vincent have not been reported in the literature to date. Consequently, we summarise events and correspondence of relevance to our discussion in Figures 3 and 4.

By the 1970s, there was capacity to monitor and anticipate changes in behaviour or the increased likelihood of the start of an eruption. Furthermore, local aid needs were defined by a locally elected government. However, preparedness remained a very poor cousin of ‘relief’. The response to the 1971–72 effusive eruptions of La Soufrière graphically illustrates this failure, even when a volcano was, in this instance, erupting and precautionary evacuations of the population had taken place (see Figure 3). With the temperature and disruption rising in the crater lake, a UK Royal Navy frigate was despatched to facilitate a short‐notice mass evacuation; it was never needed, but how this cost was covered was the subject of lengthy wrangling (see Figure 3). Basic supplies and shelter for the evacuated population were not available, and the scramble to determine who was responsible for this and why is evident in the Foreign and Commonwealth Office (FCO) records summarised in Figure 3 (despite Saint Vincent still operating as a state under ‘grant‐in‐aid’). This caused unwelcome delays in preparing for evacuation and limits to supplies of food and medicines in particular (see 22 December in Figure 3). The fixation with the need for an emergency rather than pre‐emptive measures was clear, before, during, and after evacuation (see Figure 3 and Appendix 1).

While needs were locally defined, the imperative around donor wishes remained evident here in a reluctance to create a precedent for aid in other countries (see 17 December in Figure 3), coupled with a desire to retain oversight of other donating agencies and a strong desire to be seen to be generous. This led to a lack of preparedness and confusion. The situation was politically manipulated (see February–June 1972 in Figure 3) and time was wasted on debating whether a passively erupting volcano did or did not conform to established procedure—further evidence is presented in Appendix 1.

The second eruption of the 1970s was sufficiently close to the first that political, scientific, and social memories were retained. There is also some evidence of lessons being learned between and across institutions during the crisis (see Figure 4), such that aid‐based responses were rooted in learning from 1971–72. The incumbent Premier, Milton Cato, happened to be the same individual as in 1971.

In this instance, furthermore, there was a wider understanding of other sources of funding, although they were regarded with scepticism with respect to their effectiveness (see 1 May in Figure 4) in meeting the needs of the population. And the volcanic behaviour facilitated rapid decision‐making: unrest rapidly escalated into an explosive eruption, prompting some spontaneous community evacuation (13 April in Figure 4). Nonetheless, the success of the evacuation and the lack of casualties averted a ‘disaster’; as a result, there were soon signs of a withdrawal of support, particularly naval support (both from the UK and the United States).

The provision of the long‐term rehabilitation necessary in the wake of such a protracted crisis remained troublesome (see 15 May in Figure 4). This time, the politicisation of the aid response not only manifested on Saint Vincent, but also in the general election in the UK, and there was a rapid shift in attitude towards long‐term support when the new government formed. Promises of further support were abruptly truncated and swift arrangements were made to facilitate Saint Vincent's independence, distancing the UK government from the need for further spending.

The emphasis in British correspondence throughout is on the diplomatic appearance of spending well, as much as on actually spending well, *meeting* the efforts of other aid agencies with a sceptical and critical eye (see Figure 4), while continuing to engage in inter‐departmental wrangling over responsibilities to do something effective. However, long‐term views emerged from agencies such as the Red Cross (see the Appendix) and via the experiences of UK officials on Saint Vincent (see 15 May in Figure 3).

This emphasis on being seen to be effective and spend well discourages the more ‘hidden’ support inherent in strengthening preparedness and monitoring efforts (even in the absence of a high‐impact hazard event) and encouraged visible and ostentatious responsive aid spending. This theme is well described in the context of disaster recovery and slow‐onset disasters (see, for example, Boston, Panda, and Surminski, 2020), and we will develop it further in a later paper on the capacity of volcanoes to ‘threaten’ disaster.

Some 16 years after the 1979 eruption of La Soufrière, and only six years after the devastating local impacts of Hurricane Hugo (September 1989), the start of the long‐lived eruption of the Soufrière Hills Volcano on Montserrat on 18 July 1995 demonstrated very little learning from the 1971–72 and 1979 eruptions. This was particularly the case in terms of the effectiveness of the response of the British government, but also more generally across the region.

#### The early stages of the eruptive crisis, Soufrière Hills Volcano, Montserrat (July 1995–December 1997)

**Figure 5:**
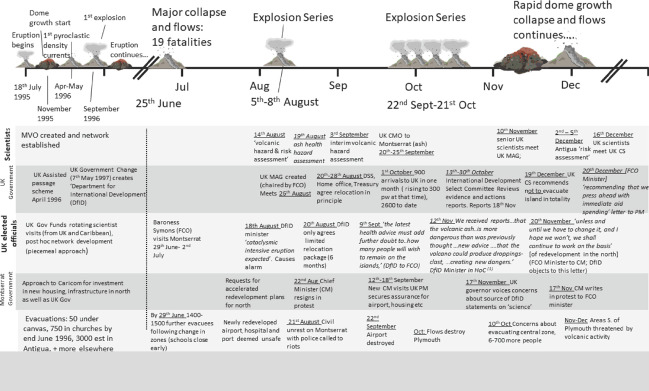
Aid activity and response, Montserrat, July 1995–December 1997 **Notes**: All materials and timings sourced from original letters and correspondence in UK National Archive files PREM 49/137 and 49/138 and contemporary scientific descriptions (MVO (Montserrat Volcano Observatory) Special Reports). (1) House of Commons Debate, 12 November (House of Commons, 1997). Abbreviations are as follows: FCO—Foreign and Commonwealth Office; DSS—Department for Social Security and Pensions; DfID—Department for International Development; PM UK—Prime Minister of the United Kingdom; PMS—Private Secretary to the UK Prime Minister; and CM—Chief Minister on Montserrat. **Source**: authors.

The activity and the contemporary response to the eruption in 1995 have generated a plethora of academic papers and grey literature (Aspinall et al., 2002; Kokelaar, 2002; Donovan, Oppenheimer, and Bravo, 2012; Hicks and Few, 2015; Wilkinson, 2015) and published parliamentary accounts (see, for example, House of Commons, 1997; UK National Archive files, PREM‐49_37 and 38), on which we draw here, with patterns of activity and response summarised in Figure 5. A 1999 evaluation of the UK government's response judged it a ‘*qualified success*’: relatively few people died and there was no malnutrition among evacuees, although conditions were crowded and actions were slow, exacerbating pre‐existing inequalities and vulnerabilities (Clay et al., 1999; Hicks and Few, 2015; Wilkinson, 2015). The failures identified here are a repetition of those identified in earlier eruptions. In particular, support for the SRC and its assessment of both risk and the need for monitoring was simply not supported, not allowing, therefore, ‘*the volcanic risk to be anticipated and then effectively monitored*’ (Clay et al., 1999). To make matters worse, there was a continued lack of willingness after the start of the eruption to invest for the longer term in monitoring, as well as, this time, confusing and contradictory lines of communication between various different scientists, present on the island at different times (House of Commons, 1997) (see Figure 5).

Beyond scientific need, the issue regarding the response centred on whether the initial crisis and actions required were ‘emergency aid’ or budgetary aid designed to help with longer‐term economic growth (see the Appendix). The identification and dispersal of aid to an Overseas Territory was perceived by the UK government as its responsibility, and part of the lack of responsiveness was attributed to the extreme uncertainty in anticipating eruptive impacts. This situation was further compounded by a change of administration in November 1996 (Montserrat) and May 1997 (UK), which led to a change in the responsible department: from the Overseas Development Agency of the FCO to the Department for International Development holding the budget and the FCO many of the decision‐making powers. As an Overseas Territory in 1997, answers or agreement needed to come, at one point, from some eight UK government departments before decisions could even be sent for approval and discussion with the Government of Montserrat. Opportunities for rapid response were obviously minimised. However, the UK administration still found time to comment on and try to influence opportunities for aid from other donors (see, for example, Figure 4 for another case in point). At heart, these problems were the same as those for the Saint Vincent eruptions some 16 years earlier, and very few of the investment decisions made to rebuild following Hurricane Hugo were robust to the infrastructure needed to counter the impacts of the Soufrière Hills Volcano (see Figure 5).

The lessons from earlier eruptions suggest that hesitation in the face of scientific uncertainty was a driver of the ineffective aid response. These lessons were not learnt or enacted during the crisis of 1995. Equally, none of the dissections of the Soufrière Hills Volcano eruption fully illuminated the interdependencies or similarities between the impacts of differing threats or highlighted the need to match the timescales of scientific uncertainty with appropriate timescales of response in terms of aid. The next section explores how durable lessons emerging across time periods could help to improve response in the future.

## Discussion: durable lessons for policy and practice that impact capacity to cope during volcanic eruptions

Effective disaster relief needs to be deployed in such a way that it maximises its influence in the immediate moment, allows affected societies to continue to respond to escalating or cascading problems, and aids recovery and rebuilding in a way that limits issues in the future. While it would not be fair to apply this modern understanding of disaster relief to a critique of individual events and past efforts at response, we use it as a means to consider the extent to which past views of disaster aid, and attendant social and political prejudices, acted to accumulate future risk. It also helps with uncovering any characteristics of empirical responses that did assist in reducing immediate or future impacts.

Volcanic eruptions represent a helpful end component of several important features of hazardous events in creating disaster. First, volcanic eruptions *threaten* disaster, with that threat dissipating or amplifying over uncertain timescales in uncertain ways. Second, when eruptions happen, they present a wide range of possible (multi‐hazard) impacts, again over uncertain timescales. These characteristics exert considerable influence over decisions made to preserve both lives and livelihoods and thus provide an ideal archetype to examine in detail and to draw parallels with many other types of hazard events. This analysis largely focusses on bilateral aid, but again, this provides the backdrop against which to consider how it also assisted or hindered the development of effective multilateral and voluntary mechanisms, as well as which groups influence decision‐making powers and hence the effectiveness of that aid.

### Longitudinal impacts: the creation of enduring vulnerability through bilateral aid

As financial aid was granted either via petition or access to state‐collated ‘funds’, the occurrence of overlapping initiatives caused some confusion and created the possibilities for those with access to power to use this for financial gain, to the detriment of more vulnerable populations. For example, following the eruption of 1902, estate owners continued to access the ‘Hurricane Loan Special Fund’, as well as the ‘Soufrière Relief Fund’ (Cameron, 1903). Furthermore, the longer‐term ‘Land and Road Relief Fund’ (see West India Royal Commission, 1898) also contributed to the rebuilding of private infrastructure.

During the eruptions of the 1970s, the position of Saint Vincent as a nation already in receipt of grant‐in‐aid acted to slow down the colonial response to the volcanic crisis. This practice of responding after the event is consistent with the time, but the asymmetry of response and recovery (rapid onset with acute need followed by long tails of recovery and debt repayment) and the need for contextualisation with other colonial commitments, meant that a response to any one individual event was always impacted by other recent events or creators of disaster. This was particularly evident in the context of the Caribbean.

The topography generated by the young volcanic islands also created the setting for a distinctive set of historical vulnerabilities to all of these hazardous events, generating both physical (high levels of exposure to multiple hazards) and social (those in the most physically vulnerable areas tended to be those with the least agency or capacity to cope) vulnerabilities. Most notably, in the ceded territory of Saint Vincent (see Table 1), the indigenous population was ‘awarded’ limited territory that was a physically vulnerable, socially remote, and economically less valuable place in which to live in 1763. Sustainable livelihoods are still challenging to this day in these areas. In addition, the practice of reserving land for large plantations of single crops continued well beyond emancipation. From the 1830s, those unwilling to live and work on plantations were also forced on to marginalised land (more remote, less productive, and prone to the impacts of multiple hazards) either on coastal strips or in the rugged interior of the islands.

In the twentieth century, issues concerning worker's rights and these commercial activities materialised during unrest and eruption on Saint Vincent in both 1971 and 1979 (see the Appendix) and emerged in Montserrat in 1996 and 1997 in terms of the right to abode and access benefits in the UK. When the need to rebuild on Montserrat arose in 1996 and 1997, issues of land ownership quickly manifested in identifying suitable places to do so, pushing cost and political issues ahead of best placement relative to long‐term mitigation of hazards (see Figure 5). Thus, systemic vulnerabilities were created via historical land use practices, combined with the relentless impacts in more marginal settings of multiple hazard events. These vulnerabilities are the outcome of centuries of colonial governance, itself indentured to the practices of a free‐market economy.

### The compartmentalisation of hazard events: consequences for additive and cascading impacts

Using volcanic eruptions as an example, we have demonstrated the extent to which damaging hazard events in the Caribbean rarely occur outwith the envelope of response and recovery in the wake of other hazardous events, and typically within a background of longer‐term economic or social crisis. Figure 2 highlights ‘index’ events for these eruptions. By this, we mean surrounding events that affected the efficacy of response or amplified the effects of the event itself (or vice versa).

We show here that, without exception, the uncertain early stages of the volcanic eruptions explored tended to involve a population and economy recovering from another recent (past few months/years) acute or political event set against a backdrop of frequent incremental losses from less intense hazards and endemic poverty associated with poor governance.

The physical outcomes of events such as intense rain or windstorms tend to be damaged vegetation, mobilised soils, and unstable ground. These have strong similarities to those outcomes from tephra fall and flows from volcanic eruptions, or ground shaking due to strong earthquakes, so it should make sense that the impacts of concurrent events will exaggerate or amplify one another. For instance, in the case of Saint Vincent, the indigenous population was in the process of negotiating a move to more stable ground following severe landsliding during the 1898 hurricane when pre‐eruptive unrest caused them to abandon their settlements prior to the climactic 1902 eruption, as land destabilised further, albeit via a different generative mechanism. Yet, in the academic literature or historical accounts, no mention could be found of these relations until the Soufrière Hills eruption, and then largely in the context of eruptive longevity and lahars (Barclay, Alexander, and Susnik, 2007) or impacts on mammal and bird populations. In historical accounts, these impacts are largely described either financially or in terms of cumulative infrastructural impacts, rather than as a consideration of causative forces, or relationships between event outcomes.

This is partly compounded by the lack of a framework to consider multiple hazards in this way, even within the context of hazard science (see, for example, De Angeli et al., 2022). There is a rich and emerging literature that analyses hazards alongside one another, or as additive functions (such as identifying land with multiple susceptibilities, but a rather smaller pool that considers them holistically in terms of the dynamic or cascading impacts on the society and environment with which they interact (Wang, He, and Weng, 2020). In turn, this invites separated protocols for mitigation, response, and preparedness for each hazard, with the momentum for change being the most recent emergency and its associated hazard. The response demands decisions that are perhaps made too quickly to embed preparedness for or mitigation of other events. More recently, however, ideas have emerged that short‐circuit this loop, such as by considering mitigative or planning measures that could generate ‘co‐benefits’ during the course of multiple hazard events (see, for example, Wilkinson et al., 2016). Our analysis shows that this requires their identification in advance, and established pathways for implementation when opportunities arise. This almost always entails listening to and understanding hazard outcomes from the perspective of the affected population, a facet often missing from or only glimpsed at in the historical record.

### The political compartmentalisation of aid for disaster (bilateral aid)

Our study reveals, too, that long‐term inadequacies in colonial governance and the political and social inequalities it created were always exerting a longer and significant influence that impacted response. Here, we have explored political influences and complexity in short‐term response. Table 2 lists the cooperating entities needed to approve financial aid for each focus eruption.

Table 2 demonstrates the proliferation of complexity, through time in terms of agreeing and releasing a response to a hazardous event, and the compartmentalisation of budgetary entities that might implement strategic investments that lead to improved planning and mitigation to reduce risk.

The complexity of these processes and this compartmentalised decision‐making demand the creation of a more simplistic mechanism to deal specifically with the emergency. This is illustrated here by the ‘Mansion House Fund’, which was used in the nineteenth century for the UK and other events in the Caribbean and is echoed in the twentieth‐century existence of emergency departments within the FCO and dedicated multilateral response mechanisms (see Table 2). However, although these agencies enable a rapid response, their focus is necessarily on immediate short‐term actions. Mobilisation requires a ‘disaster’, and responsibility wanes at the moment of learning, mitigation, and prevention, as evidenced by issues with expenditure of the Mansion House Fund and long‐winded wrangling about actions in response to the slow‐running 1971 eruption of Soufrière Saint Vincent, and culminating in the inadequate response to the eruption of the Soufrière Hills Volcano on Montserrat. Furthermore, in the presence of a department focused on emergency response, other departments are disincentivised to consider preparedness and mitigation as part of their remit, including longer‐term projects that specifically spotlight the lowering of vulnerability in other ways as part of a development agenda.

The main conclusions of the evaluation report commissioned by the UK government in response to the inadequacies pertaining to the Soufrière Hills Volcano eruption (Clay et al., 1999) echo those apparent from our analysis of responses to earlier eruptions: the need to respond using aid strategies for both the short (days and weeks) and long (months and years) term and the provision of aid driven by the needs of the recipient, not the donor. However, these recommendations cannot be met without rapid investment and decisions to release money or ‘aid’, *coupled* with aid that prepares for disasters in the future, and removes the vulnerabilities introduced by the current event.

These lessons could have been learned in the aftermath of any one of the significant happenings we have described here. The articulation of the same points evident from earlier eruptions begs the question as to whether these lessons are merely articulated or actually *learned*. The closely spaced eruptions of the 1970s on Saint Vincent demonstrated some local learning in relation to action and response, but there is rather less evidence that these exerted any influence on the subsequent response of the UK government and external donors to the early stage of activity at the Soufrière Hills Volcano. This was undoubtedly compounded by the British tradition of moving FCO officials regularly from country to country to avoid complacency. This learning is frequently articulated in the literature dealing with the political economy of decision‐making as ‘triple‐loop’ learning (Wilkinson, 2015), and permanent change is articulated with regard to the ‘policy window’ for realignment of political processes in the corrective aftermath of a disaster.

The reality here shows that learning cannot be implemented until the temporal and spatial timescales of (multiple) hazard processes and impacts are aligned with the timescales associated with political decision‐making on spatial and fiscal scales, which align with projects ranging from major infrastructural investments to local‐scale preventative and preparedness actions. By this, we do not suggest that these operate on similar spatial timescales, but that thinking around hazard analysis can be adjusted to consider these decision‐making pressures and the time frames over which they occur (that is, assess hazards together) and in turn, that financing of disaster aid widens the emphasis on long‐term support beyond the disaster moment.

This latter point is not new, but our analysis demonstrates in particular the benefits of the accumulation and exchange of knowledge as a continuous process across hazards and the benefits of adequately resourced monitoring networks, and the valuing and sharing of experience. A specific challenge in this regard is the coincidence of major hazards with rapid change during a political cycle (reorganisation or change). Both the Soufrière Saint Vincent eruptions in 1971 and 1979 and the eruptions of the Soufrière Hills Volcano in 1995–97 coincide with a major change of government, leading to delays in or alterations to aid administration.

### Into the right gaps? Prejudice, personalities, and external actors (multilateral and voluntary aid)

We have shown that, over time, disaster relief has been articulated more clearly with respect to the needs of those most affected, but that this has been offset by increased bureaucracy and continued opportunities for political interference. The final dimension of our analysis here is to consider the spontaneous successes of disaster aid evident in the historical record despite these structural issues. These often, although not exclusively, manifest themselves around voluntary aid. In the Caribbean, this type of aid is said to occupy the gaps and groupings left behind by state‐based aid mechanisms, and so looking to patterns of aid movement in this area might suggest where smaller initiatives might act to disproportionately reduce vulnerabilities to further risks. A strong challenge with this is finding these instances in the written record examined. Nonetheless, there are some positive local instances that offer templates for different ways in which to distribute aid. The network of the Commonwealth (not officially defined but broadly other countries in the British Empire at any one time during the eighteenth and early nineteenth centuries) were inclined to provide not just financial assistance but also goods of relevance to victims. In particular, the island‐to‐island practice of sending on seedlings and plants to ensure the rapid replanting of not just plantations but also subsistence foods was widespread in the early twentieth century. This had a particular association with some late nineteenth century long‐term development initiatives designed to address chronic poverty and vulnerability following a multi‐island review by Morris (1897). These created botanical stations designed to experiment and understand best practice in both large‐ and small‐scale agriculture. Intriguingly, the specialised reports pinpointed the importance of small‐scale diversification of farming practice, and a worthwhile outcome was the sharing of plants suitable for small‐scale farming following hazard events (after hurricanes and the Soufrière Saint Vincent eruption of 1902–03). This view did not prevail, as each time market economy monoculture was favoured (see, for example, Barclay et al., 2019a), and, as detailed above, there were considerable differences in 1902 between those who farmed the land and those who administered it, apropos of the primary purpose of cultivation. Morris (1897) himself attempted to intervene and requested the conduct of further experiments on the ash‐laden soil to understand best which crops were able to thrive under these conditions; this was resisted by Governor Llewelyn on the grounds that it would be a poor investment of the Eruption Fund should further eruptions occur (Blue Book, 1903).

In a similar vein, the 1999 evaluation of responses to the SHV eruption recognised that, when undertaking reconstruction projects, ‘[o]verall, construction and adaptation using local materials, know‐how and labour appear to have been more cost‐effective than solutions based on the importation and assembly of prefabricated structures’ (Clay et al., 1999, p. 60). In particular, multiple participants at a STREVA (Strengthening Resilience in Volcanic Areas) workshop remembered the positive moment of using a small amount of aid money to pay local labourers to remove and redistribute heavy ashfall in the north of the island in 2003.^3^ This example is a useful counterpoint to the stereotypes and complaints that emerged in the aftermath of the 1902–03 eruption on Saint Vincent when labour was enforced (either on plantations or via migration) and consequently resisted. And it demonstrates the benefits of aid deployed not only to respond and recover, but also in a fair and beneficial way to those employed. Collectively these insights point to the value of crisis aid driven by the context of long‐term development concerns, something that can only be achieved by embedding the concerns of the local population in those responses. Initiatives driven by donor needs and perceived as opposed to actual insights into what will help are less successful.

## Conclusion

Our study drew on the historical record of four past eruptions on the Caribbean islands of Saint Vincent and Montserrat, augmented by an analysis of other hazards and longer‐term political and social processes, to examine the role of disaster aid in alleviating short‐term problems and its contribution to long‐term vulnerabilities. We found that there are several enduring lessons that transcend the different eras in which these eruptions took place. Systemic vulnerabilities to natural hazards are created by their impacts, inadequate aid responses, and longer‐term chronic problems created by weaknesses in governance and financial management of the islands. We found, too, that the effects of the hazard are compounded by these longer‐term problems, and that in the Caribbean, most major hazards happen in sufficient spatial or temporal proximity that the cycle of response and recovery to one is broken by the occurrence of another.

Our analysis also provides new evidence on what approaches might work in the future. First, a more detailed consideration of the hazards in terms of their integrated impacts and the ways in which they amplify and affect one another would pinpoint preventative or mitigative measures that can be enacted on timescales that match political decision‐making processes. Second, by incorporating local knowledge in understandings of both hazard and risk, and in determining the effective use of aid resources, useful interventions can be identified that have a disproportionate bearing on vulnerabilities and preparedness. This has largely been illustrated by its absence from the historical record, its lack at the root of failure, and the dissent described here. Third, there is a need to decompartmentalise the way in which aid is viewed in the context of hazards. Aid for long‐term development needs to be coupled with emergency funds, with desirable shifts in infrastructure and practice preidentified for integration into rebuilding schemes when the requirement occurs.

## Appendix: list of archival sources

This appendix contains public sector information licensed under the Open Government Licence v3.0. The names of government officials have been removed and replaced with their job titles or departments.

**1812 eruption: manipulation of aid for personal benefit**

A letter from the Governor to the Administrator of the Windward Islands suggests the motivation for this. According to the letter, the colonial government wanted to purchase 132 acres of the Grand Sable estate (owner Thomas Brown) to rehouse displaced enslaved persons and turn the location into a village (township). The Governor described resistance by Thomas Brown to surrendering those lands. The estimated losses recorded are likely financial ‘persuasion’ for the selling of this land (Brisbane, 1813):

*‘Understanding however that it was the intention of Col. Browne's attorneys to make every resistance in their power to the rights of the crown and having been readily informed that of them (Mr Cayley) had declared his determination to die upon the [sic] than permit the execution of my orders, and considering that for the number of the negroes (six hundred) and [sic] their power would be insufficient for the purpose in case the parties should proceed to extremities, [sic] a letter a [sic] whereof is contained in the enclosure number 5: to Major General [sic] the officer commanding His Majesty's troops in this island, in reply to which received the answer a copy whereof is contained in the enclosure number 6‘*.

*‘In the meantime however having received information that the parties did not intend to make further resistance, I addressed to Major General [sic] the enclosure number 7; which fortunately saved the troops a long and [sic] march; and on the morning of the 20^th^ my orders were carried into effect; the first stone of the intended town was laid, and the surveyors were left to complete their survey which has since been done, as a report of a very different nature may be made of this transaction to your Lordship, I have judged it right to state the facts fully to you as they occur’*.

**1902 eruptions: perception of landowners of themselves as key victims of the eruptive impacts**

The estates of Alexander Porter were physically and economically at risk due to the large area of land exposed to the volcanic hazards. Despite being at risk, Mr Porter had a lot of wealth, which brought with it power. As an absentee learning that his estates were affected, he wrote a persuasive letter to seek compensation (Porter, 1902):

*‘… without any assistance [my] estates notwithstanding my heavy losses and doing all [fre]quently averting a crisis among the excitable negroes population especially in the Carib Country districts … I feel in face of an estimated loss of £27,000 on my estates caused by this terrible calamity that unless I receive a moderate amount of compensation … the difficulties of the government will be greatly increased with a large unemployed negro and coloured population clamouring for food’*.

**1902 eruptions: evidence of pressure to migrate for labour**

*All correspondence here relates to the original files and enclosures in the National Archives associated with the publication of the Blue Books* (1902, 1903)

Governor of the Leewards Island's correspondence of 9 January 1903 states:

*‘you will have gathered from Captain Youngs’ reports that the people in St. Vincent … have obstinately refused to listen to any proposals offered by Captain Young and myself to emigrate anywhere. I found in September, on the occasion of a visit to St. Vincent, a Petition from certain persons expressing their willingness to go to Jamaica, but before any definite proposals, such as I have just received from the Governor of Jamaica in his letter of the 30th ultimo could be laid before the people the agitators against the scheme set to work to prevent it, and it has been impossible for the Government to convince ignorant people, against the advice of their clergy, of any prospective advantages.… I am afraid the people do not wish to go anywhere they will have to work. In their native island they can eke out an existence, and seem perfectly content to live under such conditions.… The charitable assistance of the world is thrown away upon such characters'*.

About this time (11 January 1903), the Carib population of Owia and Fancy tried to bypass the local administration and directly petitioned the King in the UK about conditions in their temporary lodgings. It is rare to see direct views of this group expressed in the National Archives, although they were mediated through local clergy, as observed by the Governor's Administrator: ‘*the cover letter is in the handwriting of the Rev T. Huckerby, Wesleyan Minister of Chateaubelair, and the date on the petition is in the Rev J.H. Darrell's writing*‘. They complain further about the imperative for migration:

*‘After being huddled together for six months in the building referred to, in which we suffered greatly from overcrowding, the Governor ordered us to emigrate, which we declined to do so, St Vincent being our homeland, and that of our Carib ancestors, and there being abundant room and labour for us therein’*.

For example, in the original correspondence destined for the Blue Book at that time, a passage marked in red pencil as ‘omit’ details the Governor requesting that working‐age males be denied access to shelter accommodation on the island should they resist migration for labour offered on other islands as an ‘encouragement’ to comply. Unfortunately, this top‐down approach to handling the Soufrière Eruption Fund meant that Llewelyn ran out of ideas on how to spend the money, and GBP 25,000 was returned to the UK government with many of the needs of the refugees unmet (Pyle, Barclay, and Armijos, 2018).

**1971 eruption: the experience of the UK FCO and the impact of frequent movements**

*Material from: TNA FCO 63/881 File ANV 11/2 Part A. Caribbean Dept, HM Diplomatic Service (correspondence 6^th^ November until 5^th^ December); TNA FCO 63/882 Volcanic Activity in St Vincent File no AN V 11/2 Part B. 1971 and TNA FCO 63/883 File ANV 11/2 Part C. Caribbean Dept, HM Diplomatic Service (correspondence 5^th^ December to end December); and then FCO 63/1022 AN V 11/1 1972 (all 1972 correspondence)*.

**6 November: Deputy British Government Representative Saint Vincent (DBGSVG) to other Caribbean departments:**

*‘My only previous experience of natural disasters were the Ionian Islands earthquakes in 1953 when it was laid down that no difference was to be made in our relief efforts between British subjects and others. I believe that this is the policy we must follow here should disaster happen. We are a part of this community, and if we are not [to] be discredited both as individuals and as a group [we] must share the experiences of the people among whom we live and work’*.

**1971–72 eruption: evidence on hesitation in the provision of aid for needs pertaining to the disaster in process**

Between 1 and 15 November 1971, there was evidence of considerable unrest, and observations of crater lake warming and discolouration in La Soufrière, accompanied by seismically detected unrest.

**12 November, DBGSVG to other Caribbean departments and Caribbean Department, FCO:**

*‘London will no doubt pronounce on the question of principle … on the policy to be adopted in the event of any relief operation. Subject to their comments, I would imagine that it would be right in the event of any evacuation to give priority to our own citizens; but that otherwise the relief effort should be directed without discrimination over nationality’*.

By 15 November 1971, a lava dome was observed growing in the crater. At this point, the volcano is ‘in eruption’. On 23 November, HMS Berwick was ordered to ‘sail at maximum speed’ to Saint Vincent.

**DBGSVG to Caribbean Office, FCO:**

*‘I hope that there will be no question of presenting a bill to St Vincent government for any assistance we are able to render. This state is still under “grant‐in‐aid” and receiving very substantial development aid from UK. Eventually in one way or another the UK will no doubt accept this expenditure. Grateful if we could avoid any future difficulty by clearing this point now'*.

**24 November, the UK FCO to Deputy Under Secretary (Dependent Territories), FCO:**

*‘It soon emerged that the main difficulty facing us was finance. MOD could provide anything that was needed at very short notice, and could fly it out in a VC10 or a Hercules. but the cost of a Hercules flight alone would be over £8,000. For their part FPAD [Finance Policy and Aid Department], who have authority to provide up to £10,000 for any one emergency, cannot act until a disaster has occurred’*.

**26 November, FCO Finance Policy and Aid Department to Finance Department, FCO:**

*‘The FCO Natural Disasters subhead permits us to offer first aid relief after a disaster has happened. Similarly the ODA [Overseas Development Administration] can give aid for rehabilitation or reconstruction at a still later stage. But we are concerned at present with precautionary measures; apparently we are without financial authority to meet the cost of e.g. helicopter flights carrying vulcanologists on visits of inspection. Pre‐disaster planning is normally the responsibility of the government of the country concerned. St Vincent is an Associated State, is self‐governing except in the field of defence and external relations. But its resources are small. It has no defence forces. The UK can hardly recover the cost of assistance given in taking common sense precautions and particularly not, perhaps, if the help is given by our defence forces.… Perhaps the answer is that the cost of assistance with pre‐disaster planning should be taken into account when fixing the amount of budgetary aid to be given to St Vincent. This is a matter for ODA’*.

On 30 November, volcanologists arrive from the US and perform an overflight using infra‐red, after which they provide a briefing to the US Embassy and then head to Saint Vincent (1 December). Discussions in FCO departments continue, as the dome grows, on the validity of providing funding.

**3 December, Finance Department, FCO to Finance Policy and Aid Department, FCO:**

*‘Our Natural Disasters subhead is quite clearly entitled “Assistance after Natural Disasters Overseas” so it cannot cover planning before a disaster occurs’ (emphasis in original)*.

Meanwhile, the DBGSVG wrote to all FCO departments to advise them that an evacuation had been decided upon based on discussions on new evidence from the US volcanologists. A flash telegram was sent on 4 December to confirm this and to provide a prospective date for the announcement and enactment (7 December).

**4 December, DBSVG to Caribbean Department, FCO:**

*‘[T]he people at the centre [in Saint Vincent] have realised that the difference between absolute disaster and a more serious set‐back is measured by the amount of hard work done now’*.

**7 December 1971, a letter from the Caribbean Department, FCO to other UK government departments:**

*‘As you know the special fund upon which FPAD can draw to provide a contribution from HMG [Her Majesty's Government] towards the cost of providing relief measures in the case of natural disasters say, under present arrangements, can only be used when such a disaster has occurred … as soon as the volcano erupts we shall be ready to provide naval assistance and that they can then expect a substantial contribution from HMG to the relief fund; but nevertheless the absence in the meantime of any immediate financial or material contribution from Britain will become increasingly obvious and embarrassing’*.

One should note here the use of embarrassment as a motivation to unlock funding. On 8 December, the evacuation begins, involving many agencies and non‐governmental organisations, including the Red Cross at this point. It is not perceived as proceeding smoothly.

**9 December 1971, a letter from the Caribbean Department, FCO to the DBGSVG:**

*‘It has struck us that, amidst so much relief assistance already being given by charitable bodies and other Governments, the absence of any financial help from HMG may soon become embarrassing even if it is not already so. For your own information we are therefore pressing for the relaxation of the “no eruption, no payments” rule which governs the operation of the FCO's Natural Disaster Fund, with the idea that you might be authorised to offer the St. Vincent Government some small but respectable looking sum of money as soon as you can.‘*

**13 December 1971, a letter from the DBGSVG to FCO departments:**

*‘The Governor thinks that some actions already taken or which may need to be taken in the future, require formal proclamation of an emergency.… I went on to say that my own worries at the moment were concerned with (a) feeding refugees and (b) effects on the economy of the state. I did not see that formal declaration of emergency addendum helped much in these spheres. It was not for me to comment on internal political matters but the best answer to the opposition would be to see that the reception centres worked well and that evacuees were (a) kept happy and (b) stay fully employed. Governor said wryly that he knows his own people and that the only result of happy centres would be that others would jump on the band waggon for free meals etc.‘*.

**14 December 1971, a letter from the FCO to the Treasury:**

*‘So far, British Government help towards relieving the situation has been confined to assistance given by our diplomatic representative on the spot, and by a visiting ship, in the setting up of an operational centre; and in the conveyance by the ship's helicopter of seismographic gear and scientists to and from the volcanic crater. This hardly seems adequate in view of our “association” with St. Vincent … my purpose in writing is therefore to ask you to agree that we may authorise an immediate gift to the St Vincent Government of an amount up to £5000‘*.

Now, the provision of aid to Saint Vincent becomes a point of contention because of parity with other countries with which the UK has an ‘association’.

**17 December 1971, a letter from the Treasury to the FCO:**

*‘Although the sum at issue is minor I would be reluctant to agree to expenditure explicitly in anticipation of a disaster because of the vastly more expensive repercussions which such a decision might present in e.g. the context of flood prevention measures in India or Pakistan.… I suggest that ODA be asked to find £5000 from within the total aid programme’*.

**17 December, a handwritten note from the FCO:**

*‘Mr xxx. Can you make something out of this lemon?‘*.

**17 December, a handwritten note from Mr xxx:**

*‘Mr yyy (Caribbean Department). We discussed and you said you could take this up’*.

**22 December, a letter from FCO Aid to FCO Finance:**

*‘I am astonished that the Treasury attitude in (HM Treasury) letter of 17 December to Mr Bottomley … if the Treasury argument is that no disaster has yet occurred I think that this should be equally rebuffed. Effectively (financially) the disaster began to occur as soon as the Government incurred expenditure and began to lose revenue. Are the Treasury seriously suggesting that the relief money should be held back until a predetermined number of persons have been killed?*

**Evidence on the impact of weak preparedness and poor funding on evacuation:**

**22 December, the DBGSVG (part of a 27‐page report to all FCO offices):**

*‘If, shortly, there is an explosive eruption, the Government will be praised for its foresight, and returned to power at the next elections. But if, as is quite as likely, the present activity continues for some time the most probably development is a gradual spread of discontentment among evacuees and those who have had to suffer their presence in their communities and a progressive deterioration of the State's economy, faced as it is with reduced productivity of the agriculture of the clearance areas and with the burden of supporting the evacuees*…

*Social problems are already appearing. The evacuees, removed from their homes are housed in schools and mission halls.… But except where there is a determined and experienced campleader, public areas, kitchens and sanitary facilities are neglected. The risk of disease is high*.

*Cattle rustling has started, and animals are still being sold at ridiculously low prices'*.

**1971, political manipulation of the situation:**

With growth of the lava dome slowing, the evacuation area was changed on 5 and 6 January and some evacuees returned home. By the end of January, dome growth was very slow and only one SRC scientist remained on the island. Meanwhile, on 29 February, the Minister for Agriculture, Trade, and Tourism resigned, citing that he was opposed to the Premier's handling of the eruption issue, among other reasons. All evacuees were allowed to return home on 4 March, and the eruption was declared over on 20 March. On 8 March, parliament was dissolved, and a new administration began, with the premiership passing from Milton Cato to James Fitz‐Allen Joshua on 7 April.

**14 June 1972, the DBGSVG to the FCO Castries Administration, Saint Lucia:**

*‘[New Premier. PM SVG] and his colleagues have been industriously muck‐raking ever since the elections. Themselves highly qualified practitioners in the art of squandering public money, they are taking advantage of every opportunity open to them to accuse the [previous Premier] Administration of graft, corruption, nepotism and so on*.

*Since the evacuation the plantation has managed to operate largely because the older worked, both men and women, continued to do their jobs. But the situation at the moment is that only about half the coconuts produced can be “split” for copra‐making at Orange Hills itself. The remainder must be transported to another plantation further south to be cut open. The coconut meat is then brought back to Orange Hills for drying. There is no prospect of an immediate improvement. Indeed, the reverse is the case*.

*Orange Hills is an area which is solidly pro‐Joshua, and there is in process at the moment an operation of removing from minor government jobs, such as a road gang or truck driver, known supporters of the Labour Party. Their places are being taken by PPP [People's Political Party] support and Orange Hills has lost more than thirty of its most experienced workers during the past three weeks for this reason alone. Their acquired skills and knowledge of the plantation cannot be replaced'*.

**Learning across eruptions in the 1970s:**

For example, the UK SVG representative wrote:

*‘[the PM SVG] and his team and Police HQ [headquarters] worked hard and long hours and with unaccustomed decisiveness. It is largely due to them that the initial evacuation proceeded so quickly and smoothly’*.

*‘As the evacuation will last at least 9 weeks and probably much longer, the British Red Cross Society has sent a disaster relief delegate to the island for 2 weeks to assess the situation and to determine the need for further assistance … the volcano is still very unstable and although help has been coming in from many sources certain needs have not yet been covered. The Society has therefore asked the League to make a limited appeal to North American and Caribbean Societies’ (1^st^ May British Red Cross Bulletin in the National Archives)*.

**1979: rapid response to early explosions and issues concerning the visibility of this ‘aid‘**

*All correspondence in quotes reproduced from TNA FCO 44/2030 and TNA FCO 44/2031*.

On 11–12 April, there were first small tremors, and then a continuous tremor by the evening of 12 April, with low rumblings around midnight. Just after that, the SRC telephoned the Premier to inform him of events. On 13 April at between 04:00 and 05:00, the first residents noticed ash, and self‐evacuation on the west side began. At 05:00 evacuations were called. At 05:45, the first explosive eruption took place (series of four until 21:15).

**15 May, Deputy British Government Representative DGBR – ‘Lucas’ update:**

*‘Reception centres were set up in schools, churches and community centres and coordinators appointed to take charge of each centre. By 14 April 30 such centres had been established containing an estimated 12,000 evacuees. Further camps were gradually established to avoid overcrowding and eventually 65 were in existence containing 12,947 evacuees. An estimated 2,000–3,000 evacuees were and are accommodated in the houses of relatives and friends, but are being fed at the camps*

On 14 April, there were more explosions, and on 16 April, the UK approved an initial amount of GBP 25,000 for food and assistance with medical aid. On 17 April, there were more explosions.

*‘Also according to LIAT [Leeward Islands Air Transport Services Limited] between 2,500 and 3,000 people left St Vincent by air during the first week of the crisis’*.

**On 18 April, the British Government Representative (Barbados) visited a US Coastguard plane with supplies and then reported to FCO:**

*‘The Prime Minister seemed somewhat off‐hand when we arrived and spoke a bit scathingly about the quantity of supplies and said five tons was no good, they needed 500 tons … he seems to undertake a great deal of the administration himself even locating missing persons. People were constantly coming and going in his office and the telephones kept ringing*.

*The DGBR [Deputy British Government Representative – St Vincent] asked us to look into the possibility of having first call on some charter planes in case evacuation of British Residents should become necessary*.

*Our visit seemed to be worthwhile in that we received good press coverage on arrival at the airport and we were seen travelling through town and visiting reception centres’*

**19 April, a report from an FCO Information Officer:**

*‘it was suggested that we should restrict our efforts in the first instance to providing medical supplies since it would clearly be more sensible from an economic point of view that heavy articles should be provided from nearer at hand than the UK. The ODM [Office of Disaster Management] Disaster Unit worked throughout the weekend to gather together medical supplies specifically requested by the Government of St Vincent … in addition I received permission to spend up to £25,000 in meeting bills for emergency supplies of food, to be purchased in either Barbados or St Vincent’*.

**20 April, a news item on the BBC (British Broadcasting Corporation) television news by Martin Bell:**

*‘The island is a British responsibility but the external relief operation is very obviously American. United States coastguard helicopters fly rescue and reconnaissance missions, the Americans have made an immediate offer of £150,000 worth of emergency aid, against only £25,000 from the British’*.

The FCO sent a strongly worded (unrecorded) complaint to the BBC about the tone of this reporting.

**25–26 April, further explosions**

**1 May, an unknown FCO official's handwritten comments (in brackets) on a United Nations Press Release:**

*‘The United Nations Children's Fund (UNICEF) is flying in assistance for 15,000–20,000 people evacuated from the volcanic eruptions on the Caribbean island of Saint Vincent. The USD 6,000 worth of assistance included urgently‐needed drugs and oral rehydration salts [for use in cases of acute dehydration following gastroenteritis ????? necessary]. Conditions in the camps therefore have to be very carefully monitored to prevent outbreaks of gastroenteritis and typhoid [almost unknown in the area? Not very good 3/10]‘*.

On 3 May, following the UK general election, the government become Conservative, such that Foreign Secretary David Owens becomes Lord Carrington. Civil servants will have remained in place. No further explosions mean that the evacuation zones begin to change. On 14 May, the evacuated zone is moved to north of Chateaubelair and Rabacca only.

**15 May, BBC responds to the complaint. Ken Callaway Chief Assistant to Television News Editor:**

*‘As you say, the tenor of the report is that the British contribution to relief was comparatively small. If Martin Bell, on the spot, said so, I am inclined to believe him. He is our senior and most experienced correspondent, and not one who would lightly say anything to the detriment of his own country’*.

A handwritten note in the margin inferred from FCO member: ‘Mr Callaway as well as Mr Bell, scores 0/10. But I am not inclined to take this any further … please copy with enc (transcript) to Disaster Unit ODM, News Dept FCO’.

**1979, political machinations around response and aid, and the long‐term implications**

**25 April, memo, West Indies Department, FCO:**

*‘You may be interested to know that the Vincentian vote in this country is concentrated in Reading North, High Wycombe and Aylesbury and the Labour candidates in each of these constituencies have been pressed by immigrant organisations to state what the government has done, while opposition candidates have been accusing the Government of responding tardily to the disaster in St. Vincent’*.

**15 May, memo, West Indies Department, FCO:**

*‘I suppose one cannot blame Prime Minister SVG for trying to extract the maximum amount of assistance from as many donors as possible while the disaster is still fresh in many people's minds. He will have formidable problems later on when St Vincent is no longer in the headlines*.

*The total value of UK emergency aid to St Vincent is now in the region of £100,000 and consists of cash for local purchase of food and other supplies. In addition, three British seismologists are on island to help local experts to analyse the volcano's current activity and the prospects for the future. And a frigate will always be within two or three days steaming distance. Our main contribution will be to help St Vincent repair the long term damage to the economy*.

*And it will be the longer term that most assistance will be required. Banana shipments are down by two‐thirds, other crops have been similarly affected and although no accurate figures exist, a lot of livestock must have been lost. It will take time, perhaps years, to get agricultural production back to what it was. This years’ Carnival has been cancelled which adversely affect the tourist industry and much of the impact of the Cooper Lybrand team's effort to attract investment have been lost'*.

**1995–97 emergency on Montserrat:**

*‘When it came to projects DFID [Department for International Development] could not seem to decide whether the situation should be treated as an urgent development problem or as a true emergency‘* (Taylor, 2010).

*‘It has three pockets out of which to finance Montserrat: first, emergency aid, which came into operation because of the volcanic devastation; secondly, budgetary aid to support the budget of the Government of Montserrat, over which rigid control is exercised; and thirdly, development finance‘* (House of Commons Debate, February 1998).

Department for International Development Minister regretfully commented:

*‘we had to work within the existing machinery. You cannot change the machinery of government or its budget in the middle of an emergency‘* (Minutes of the UK Government International Development Select Committee, November 1997).

**Additional references**

TNA CO 321/214 Colonial Office. Windward Islands Original Correspondence. 1902 Letters from various government offices (departments), other organisations and individuals most of which relate to the despatches sent from the governor in CO 321/210, CO 321/211, CO 321/212, and CO321/213.

TNA CO 321/218 Colonial Office. Original Correspondence from Governor R.B. Llewellyn, January–July 1903.

TNA FCO 44/2030 and 44/2031. Foreign and Commonwealth Office. West Indian Department, Registered Files, Smaller West Indian Territories (WB and WW Series).

TNA FCO 63/881, 63/882, 63/883, and 63/1022. Foreign and Commonwealth Office. North American and Caribbean Department and Caribbean Department. Registered Filed (AN) Series.

## Acknowledgements

This paper arose in part from conversations sparked through the AHRC (Arts and Humanities Research Council) Disasters Network, particularly through the inspiration provided by Caroline Williams of the University of Bristol. We wish to acknowledge the support of the Global Challenges Research Fund (grants: AH/S00579X/1 and NE/P0175719/1) and ‘Curating Crises’ (grant: AH/W00898X/1). Jenni Barclay was also supported by a Royal Society APEX Award (grant: APX/R1/180094), and Jazmin P. Scarlett was supported by a University of Hull, Department of Geography, Geology and Environment scholarship and the Dudley Stamp Memorial Fund of the Royal Geographical Society (with IBG). David M. Pyle acknowledges the support of the NERC (Natural Environment Research Council) Centre for Observation and Modelling of Earthquakes, Volcanoes and Tectonics. Two anonymous peer reviewers are thanked for their helpful comments, which led to improvements in this paper.

## Data availability statement

The data that supports the findings of this study are available in the supplementary material of this article.

## Endnotes

2 Also see https://uwiseismic.com/about-us/history/ (last accessed on 25 May 2022).

3 For more information, see https://streva.ac.uk/forensic-workshop/montserrat-september-2012/ (last accessed on 25 May 2022).
